# Supplementary material for: Iterative improvement in the automatic modular design of robot swarms
Source: PeerJ Comput Sci. 2020 Dec 7;6:e322. doi: 10.7717/peerj-cs.322 (PMC7924708; doi:10.7717/peerj-cs.322)
Supplement: Supplemental Information 3 [file peerj-cs-06-322-s003.zip › argos3/doc/api/standalone/a00636_source.html]

ARGoS: plugins/simulator/visualizations/qt-opengl/qtopengl\_application.h Source File


- Main Page
- Related Pages
- Namespaces
- Classes
- Files

- File List
- File Members

# plugins/simulator/visualizations/qt-opengl/qtopengl\_application.h

Go to the documentation of this file.

```
00001 
00007 #ifndef QTOPENGL_APPLICATION_H
00008 #define QTOPENGL_APPLICATION_H
00009 
00010 namespace argos {
00011    class CQTOpenGLApplication;
00012 }
00013 
00014 #include <QtWidgets/QApplication>
00015 
00016 namespace argos {
00017 
00018    class CQTOpenGLApplication : public QApplication {
00019 
00020       Q_OBJECT
00021 
00022    public:
00023 
00024       CQTOpenGLApplication(int& n_option_num,
00025                            char** ppc_options) :
00026          QApplication(n_option_num,
00027                       ppc_options) {}
00028 
00029       virtual ~CQTOpenGLApplication() {}
00030 
00031       virtual bool notify(QObject* pc_receiver,
00032                           QEvent* pc_event);
00033 
00034    };
00035 
00036 }
00037 
00038 #endif
```

---

Generated on 10 Jul 2018 for ARGoS by 
 1.6.1
